# Effect of Fish Bone Bioactive Peptides on Oxidative, Inflammatory and Pigmentation Processes Triggered by UVB Irradiation in Skin Cells

**DOI:** 10.3390/molecules26092691

**Published:** 2021-05-04

**Authors:** Andreea Iosageanu, Daniela Ilie, Oana Craciunescu, Ana-Maria Seciu-Grama, Anca Oancea, Otilia Zarnescu, Ionut Moraru, Florin Oancea

**Affiliations:** 1 National Institute of R&D for Biological Sciences, 296, Splaiul Independentei, 060031 Bucharest, Romania; andreea.iosageanu@gmail.com (A.I.); danielailiee24@gmail.com (D.I.); ana.seciu@yahoo.com (A.-M.S.-G.); oancea.anca@gmail.com (A.O.); 2 Faculty of Biology, University of Bucharest, 91-95, Splaiul Independentei, 050095 Bucharest, Romania; otilia.zarnescu@bio.unibuc.ro; 3 Laboratoarele Medica SRL, 11, Frasinului Street, 075100 Otopeni, Romania; ionutmoraru@pro-natura.ro; 4 National Institute for R&D in Chemistry and Petrochemistry—Icechim, 202, Splaiul Independentei, 060021 Bucharest, Romania; florin.oancea@icechim.ro

**Keywords:** collagen peptides, free radicals scavenging, antioxidant activity, biological activity, proinflammatory cytokines, melanogenesis, lipid peroxidation, photoaging, sunscreen

## Abstract

In the present study, we evaluated for the first time the photoprotective effect of fish bone bioactive peptides (FBBP) preparation isolated from silver carp (*Hypophthalmichthys molitrix*) discarded tissue using in vitro experimental models of skin cells exposed to ultraviolet B (UVB) irradiation and stressing agents. FBBP preparation was obtained by papain treatment of minced bones and centrifugal ultrafiltration, and the molecular weight (MW) distribution was characterized by size exclusion and reversed-phase high performance liquid chromatography (RP-HPLC). In vitro assessment of the effect of FBBP pretreatment in UVB-irradiated L929 fibroblasts and HaCaT keratinocytes revealed their cytoprotective activity. Their capacity to efficiently reduce reactive oxygen species (ROS) production and lipid peroxidation varied in a dose-dependent manner, and it was greater in fibroblasts. A decrease of proinflammatory cytokines secretion, in particular of tumor necrosis factor alpha (TNF-α), was found after FBBP pretreatment of THP-1-derived inflamed macrophages. Melanin production and tyrosinase activity investigated in UVB-irradiated Mel-Juso cells were lowered in direct relation to FBBP concentrations. FBBP fractions with high radical scavenging activity were separated by ion exchange chromatography, and two collagenic sequences were identified. All these results offer new scientific data on aquaculture fish bone-derived peptides confirming their ability to control the antioxidant, anti-inflammatory and pigmentation processes developed during UV irradiation of skin cells and recommend their use as valuable natural ingredients of photoprotective cosmeceutical products.

## 1. Introduction

UV radiation is considered one of the most dangerous environmental factor that produces minor skin conditions, such as erythema, roughness, pigmentation and wrinkles, but also melanoma and non-melanoma skin cancer [1]. In 2012, The International Agency for Research on Cancer classified UV radiation as carcinogenic to humans. Studies have shown that prolonged exposure to UV radiation triggered the activation of cytokines and growth factor receptors, leading to an inflammatory response and overexpression of metalloproteinases (MMPs), such as collagenases (MMP-1 and MMP-8), stromelysin (MMP-3) and gelatinases (MMP-2 and MMP-9), involved in the degradation of the extracellular matrix and, consequently, causing connective tissue remodeling [2]. Among the three types of UV radiation, UVB is known to be responsible for cancer and other skin conditions due to penetration of epidermis and upper dermis [3]. The excessive production of ROS after UVB exposure caused changes in skin cells structure and function, cell cycle progression and accumulation of mutations [4].

Skin photoprotection can be topically or orally provided by administration of natural compounds, such as vitamins, polyphenols and flavonoids, which mainly act as free radical scavengers and antioxidants [5]. In the past few years, the protective effect of fish bioactive peptides has been extensively studied, and has been shown to inhibit MMP-1 and MMP-2 and stimulate the biosynthesis of fibrillar collagen types I and III in the extracellular matrix [6]. It was documented that the aquatic species represent a remarkable source of biologically active compounds with antioxidant, anti-inflammatory and antimicrobial properties, which make them suitable for medical and cosmeceutical use [7]. In addition, replacement of land vertebrates with marine and freshwater fish can prevent the spread of contagious diseases, such as bovine spongiform encephalopathy and swine fever, as well as respond to religious restrictions of animal protein consumption [6].

Although several marine sources were exploited hitherto [8], in the present study, the possibility of harnessing new bioactive compounds from aquaculture fishery by-products was explored, in order to reuse them in the cosmeceutical industry. Every year, millions of tons of fish are destined for human consumption, but only a small part of the waste is reused. In many countries, fish skin, head, fins, skeleton and viscera are thrown away, causing environmental problems, or, at best, they are reused as animal feed. For this reason, fish waste valorization by tissue enzymatic hydrolysis has been proposed for the extraction of bioactive peptides [9]. This method has numerous advantages, such as mild reaction conditions, enzyme specificity and control of the hydrolysis degree [10]. Many commercial proteases, such as alcalase, papain, pepsin, trypsin, α-chymotrypsin, pancreatin, flavourzyme, pronase, neutrase, protamex, bromelain, cryotin F, protease N, protease A, orientase, thermolysin and validase, have been tested in different conditions, varying the reaction parameters, such as temperature, time and pH [6,11,12]. The silver carp (*H. molitrix*) is an aquaculture species belonging to the Cyprinidae freshwater family of fishes. Studies on the enzymatic hydrolysis of its waste tissues discarded after industrial processing have mainly focused on skin [13] and muscle [14,15,16]. Papain treatment of discarded *H. molitrix* bones followed by centrifugal ultrafiltration resulted in a peptides mixture containing low MW compounds useful as dietary supplement after microencapsulation with flavonoids [17]. Preliminary data on the preparation’s ability to stimulate cell adhesion and migration in human keratinocytes culture were also reported, as the main processes during skin wound healing [18], which required further investigations.

It was previously shown that protein hydrolysates obtained from the skin of marine fish, such as cobia (*Rachycentron canadum*) [19], cod (*Gadus macrocephalus*) [20], croaker (*Otolithes ruber*), horse mackerel (*Magalaspis cordyla*) [21], pollock (*Pollachius virens*) [22] and righteye flounders [23] provided an important source of antioxidant and immunomodulatory peptides. Moreover, a study reported that tilapia fish skin gelatin hydrolysates contained a hexapeptide, which decreased ROS generation in UV-irradiated mouse embryonic fibroblasts [24].

In addition, bioactive peptides containing collagen-like sequences could exert photoprotective effect on irradiated skin and efficiency in photoaging by their ability to delay the onset of wrinkles, inhibit the involved MMPs [25] and reduce melanin synthesis as a result of tyrosinase inhibition [26]. Oral administration of Pacific cod (*Gadus macrocephalus*) hydrolysates influenced MMPs inhibition in UV-exposed mice skin [27], while that of collagen peptides from silver carp skin induced antioxidant activity in UV-irradiated mice skin [28]. In most studies, peptides with a MW ranging 100–3000 Da exerted stronger antioxidant effect, mostly due to easier penetration of the gastrointestinal and skin barrier and facilitation of cellular uptake.

The aim of the present study was to characterize the FBBP preparation isolated from *H. molitrix* to investigate the photoprotective effect using in vitro experimental models of skin cells exposed to UVB-irradiation, oxidative stress and inflammation and identify the sequence of peptides exerting high antiradical activity. FBBP cytoprotection and ROS production were evaluated in stressed L929 fibroblasts and HaCaT keratinocytes cultures; anti-inflammatory activity was analyzed in THP-1-derived inflamed macrophages; and melanogenesis was assessed in irradiated Mel-Juso cell culture, in order to investigate peptides use as valuable natural ingredients for the development of new cosmeceuticals with photoprotective and anti-photoaging effect. No studies on fish bone peptides action in HaCaT and THP-1 cell cultures were found in the scientific literature.

## 2. Results and Discussion

### 2.1. Fish Bone Peptides Preparation

Bone matrix is primarily consisting of type I collagen (90% of total protein) [29]. Pretreatment stage and papain enzymatic treatment in controlled conditions of discarded *H. molitrix* bones provided a fish bone protein hydrolysate (FBH). The following centrifugal ultrafiltration produced an opalescent solution of FBBP having 92.6% peptides content, which indicated an easy and efficient technology, recommended for scale-up. The yield of FBH preparation was 54.16% (*w*/*w*), while that of FBBP preparation was 7.08% (*w*/*w*) in a dry weight (d.w.) basis. It was documented that fish hydrolysate has generally provided low debris quantity, compared to land vertebrate collagen hydrolysates subjected to filtration, facilitating a low-cost process [29].

Several fish bone tissues have been previously processed by enzymatic hydrolysis using various enzymes to produce antioxidant peptides with different MW and sequence (Table 1). No study was found on antioxidant peptides prepared by papain-treatment of silver carp bones.

### 2.2. MW Distribution

**Figure 1:**
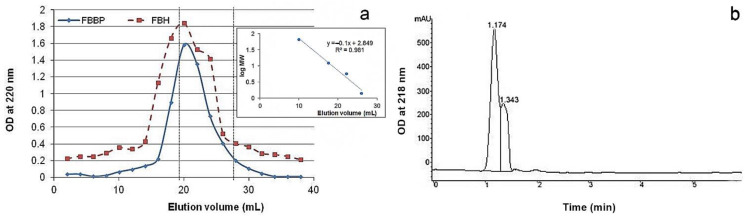
MW distribution of peptides isolated from *H. molitrix* bones determined by chromatography analyses: (**a**) Size exclusion chromatography of FBH and FBBP filtered preparation on Sephadex G-75. The insert represents the standard curve built using proteins of known MW (albumin, cytochrome c, insulin and bacitracin). (**b**) RP-HPLC of FBBP on Europa C18 peptide column.

MW distribution of peptides from FBBP preparation was analyzed by size exclusion and RP-HPLC. The chromatograms are presented in Figure 1.

The results of size exclusion chromatography showed that the ultrafiltered FBBP preparation presented a single elution peak, while FBH had a minor peak at high MW and a wide shoulder peak. However, the asymmetric shape of the FBBP peak indicated non-homogeneous distribution of peptides MW. A higher elution volume (67%, *v*/*v*) corresponded to FBBP having lower MW ranging 700–7000 Da, according to the calculations based on the standard curve (Figure 1a). These results indicate that papain treatment of silver carp bones for 6 h yielded FBH consisting of a heterogeneous mixture of oligopeptides, while the additional centrifugal filtration step led to separation of FBBP, a homogeneous fraction rich in low MW peptides.

The profile registered by RP-HPLC analysis presented a split peak at 1.174 and 1.343 min (Figure 1b). The peaks corresponding to retention times under 5 min were likely to contain small, hydrophilic fragments or free amino acids, as reported for other enzymatic fish hydrolysates [36].

### 2.3. UV Spectroscopy

**Figure 2:**
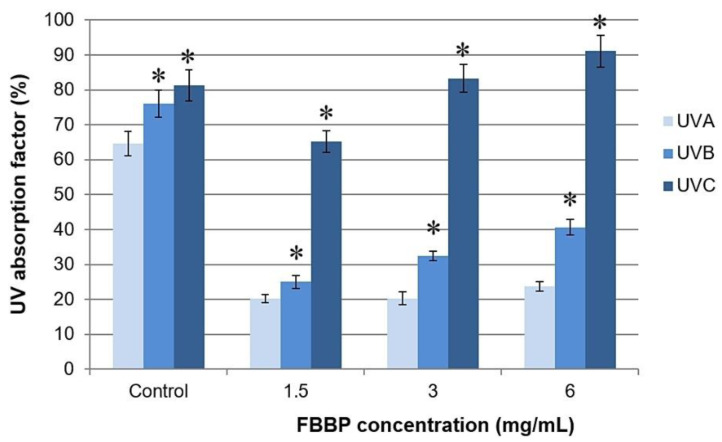
UV absorption factor of fish bone bioactive peptides (FBBP) preparation isolated from *H. molitrix* and commercial SPF6 lotion (control) in UVA, UVB and UVC domain, at different concentrations. The values were reported as percentage and expressed as mean ± SD (*n* = 3). * *p* < 0.05, compared to UVA.

Serially diluted concentrations of FBBP preparation were analyzed for the ability to absorb UV radiation by spectra registration on each UV domain, namely UVC (220–290 nm), UVB (290–320 nm) and UVA (320–400 nm). The results are presented in Figure S1. From the values of UV absorption factor, it was observed that FBBP could efficiently absorb UVC radiation (65–91%) and partially absorbed UVB (25–41%) and UVA (20–24%) radiation (Figure 2). The absorption capacity varied in a dose-dependent manner, especially in the case of UVC and UVB radiation. A commercial SPF 6 lotion used as control showed high values of UV absorption factor in all domains, varying from 81% in UVC to 64% in UVA (Figure 2).

A sunscreen provides protection on the entire UV domain (200–400 nm) due to constituent organic and inorganic filters that absorb and reflect, respectively, the UV radiation [37,38]. Plant extracts and, in particular, polyphenolic compounds, such as rutin, ferulic acid and caffeine were clinically tested as natural UV filters and provided enhanced photoprotection to traditional sunscreens, delaying erythema through different mechanisms of antioxidant and anti-inflammatory action [38]. Gelatin peptides conditioned as nanoparticles played an even more important role than rutin, enhancing the antioxidant/anti-inflammatory results, but showed no efficacy against UVB radiation [38]. In our study, FBBP preparation presented variable UV absorption, according to its concentration and UV domain. The high UVC absorption is not relevant for a sunscreen, as these rays are blocked by the ozone layer. Due to low FBBP capacity to absorb UVB and UVA radiation involved in producing erythema, solar burns or even melanoma, they could not be considered chemical filters for sunscreens. Despite of this observation, we continued the research, at the cellular level, using experimental models that mimicked the processes taking place in the irradiated skin, in order to investigate their biological efficiency and effect on the metabolism of skin cells.

### 2.4. Cytocompatibility of FBBP in L929 and HaCaT Cells

**Figure 3:**
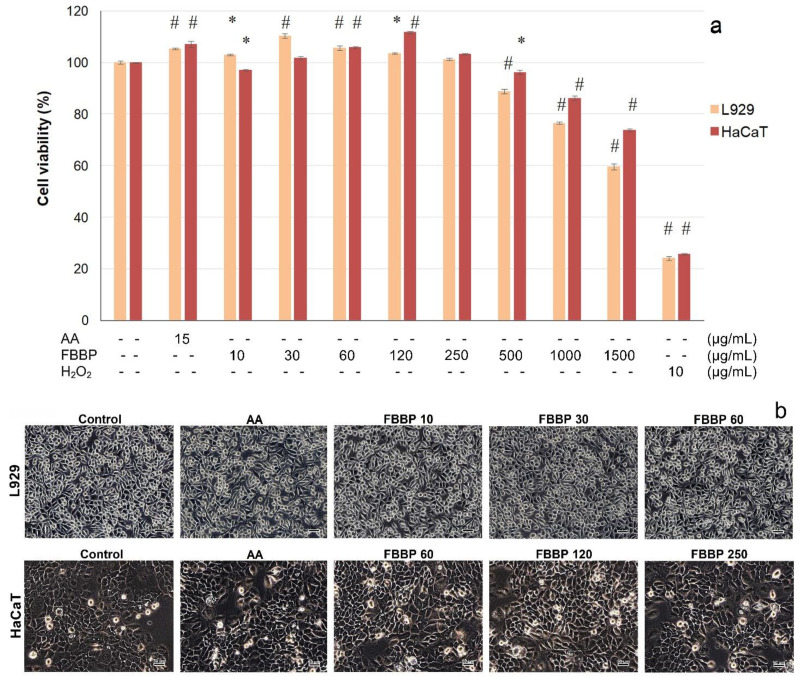
Cell viability of L929 and HaCaT cells cultivated in the presence of different concentrations of FBBP and AA at 48 h of cultivation, evaluated by MTT assay (**a**). The values are expressed as mean ± SD (*n* = 3). * *p* < 0.05, ^#^ *p* < 0.01, compared to the untreated cells (control). Cells treated with H_2_O_2_ served as positive control. Phase contrast microscopy observations of cell morphology in L929 and HaCaT cells cultivated in the presence of different concentrations of FBBP (**b**). Scale bar = 50 µm.

In vitro cytocompatibility of FBBP isolated from discarded *H. molitrix* bones was evaluated towards skin cells, in an experimental model of direct contact, according to ISO 10993-5 for cytotoxicity testing of medical devices by 3-(4,5-dimethylthiazol-2-yl)-2,5-diphenyl-tetrazolium bromide (MTT) assay and light microscopy observations. The results obtained in L929 fibroblast and HaCaT keratinocyte cultures are presented in Figure 3.

In L929 cells, FBBP presented a wide range of cytocompatibility with cell viability values above 80% in the range of concentrations of 10–500 µg/mL (Figure 3a). At higher concentrations, the cell viability decreased in a dose-dependent manner, reaching 60% cell viability at a concentration of 1500 µg/mL FBBP. It was observed that the treatment with 10–120 µg/mL FBBP significantly (*p* < 0.05) increased the cell proliferation of L929 cells, compared to the untreated control. The highest increase of cell proliferation (1.1-fold) was recorded at a concentration of 30 µg/mL FBBP and ascorbic acid (AA) (1.05-fold), a known bioactive agent with key role in skin health.

In HaCaT cells, FBBP were cytocompatible at a wide range of concentrations (10–1000 µg/mL) (>80% cell viability) and presented only a slight decrease to 74% cell viability at a concentration of 1500 µg/mL (Figure 3a). The keratinocytes proliferation was significantly (*p* < 0.05) stimulated by 1.12-fold, at a concentration of 120 µg/mL FBBP, similar to AA treatment (1.07-fold).

Light micrographs showed that cells treated with different concentrations of FBBP presented a similar morphology to that of the untreated cells, in both L929 fibroblast and HaCaT keratinocyte cultures, confirming peptides cytocompatibility with skin cells (Figure 3b). The cultures displayed the characteristic phenotype of adhered cells, spread on the entire surface, and the cell density was similar to that of control and AA-treated cells.

### 2.5. Cytoprotective Effect of FBBP in UVB-Irradiated L929 and HaCaT Cells

**Figure 4:**
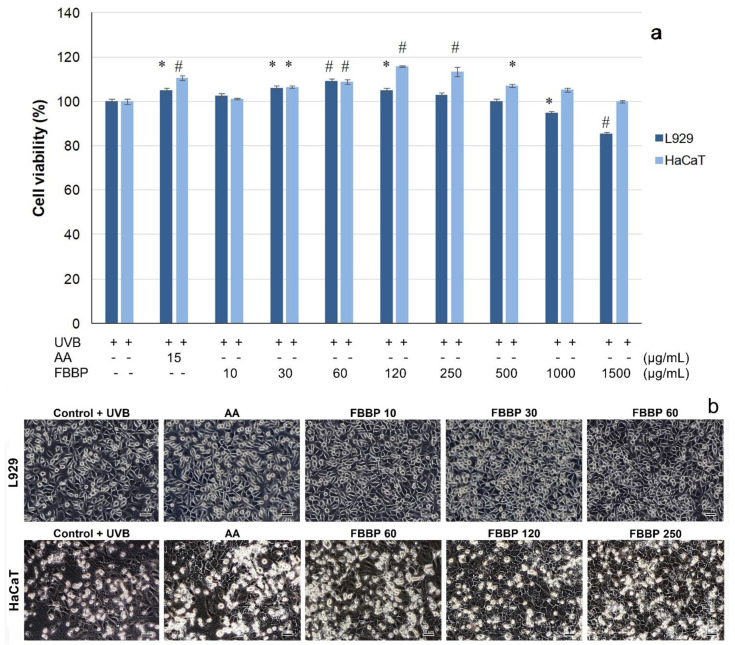
Cell viability of L929 and HaCaT pretreated with different concentrations of FBBP and AA, exposed to UVB irradiation and cultivated for 24 h, as determined by MTT assay (**a**). The values are expressed as mean ± SD (*n* = 3). * *p* < 0.05 and ^#^ *p* < 0.01, compared to UV control. H_2_O_2_-treated cells served as positive control. Light microscopy observations of cell morphology in UVB-irradiated L929 and HaCaT cells pretreated with different concentrations of FBBP (**b**). Bar scale = 50 µm.

The cytoprotective capacity of FBBP investigated in the UVB-irradiated experimental model of cultured skin cells was investigated in the same range of concentrations. MTT results show that FBBP treatment applied to L929 fibroblasts prior to UVB irradiation was able to significantly (*p* < 0.05) stimulate the cell metabolism, at concentrations of 30–120 µg/mL (Figure 4a). The values of cell viability were 1.03–1.09-fold higher than that of UV-treated control cells and similar to that of AA-treated cells (1.05-fold), at 24 h post-exposure. The maximum value of cell viability (109.21%) was recorded at a concentration of 60 µg/mL FBBP. Similarly, pretreatment of HaCaT cells with different concentrations of FBBP (30–500 µg/mL) significantly (*p* < 0.05) increased the cell viability by 1.05–1.16-fold, with a maximum value recorded at 120 µg/mL FBBP (115.88%) (Figure 4a).

Cell morphology observations performed immediately post-UVB exposure showed that irradiated cells (UVB control) presented altered characteristics with numerous globular cells in suspension (data not shown). In turn, FBBP-pretreated cells had few globular cells in suspension and the majority of cells adhered to the surface, indicating peptides capacity to protect skin cells against UVB action (data not shown). At 24 h post-UVB exposure, the morphological analysis of FBBP-pretreated cells confirmed the photoprotection of L929 cells at 10–60 µg/mL concentrations and that of HaCaT cells at 60–250 µg/mL concentrations (Figure 4b).

Similar studies showed that an antioxidant peptide of 1673 Da isolated from frog skin secretions had the ability to penetrate the cell membrane of HaCaT keratinocytes and to stimulate in vitro migration mechanisms, but also to prevent skin inflammation and photoaging of UVB irradiated mice skin by topical administration [39]. Collagen peptides from deer tendon with MW 5–13 kDa were able to penetrate the mice skin, unlike native collagen, and promoted cell proliferation [40]. In vivo studies reported that tilapia scales collagen peptides of 3.5–4.5 kDa showed better penetration of mice stratum corneum than smaller peptides (1.3 kDa) and improved women skin moisture and elasticity [41]. Studies on UV-irradiated mice fed with antioxidant peptides of 1–3 kDa from silver carp skin gelatin exerted the best protecting effect on skin structure, compared to casein peptides [28]. Moreover, consumption and absorption of fish scale collagen dipeptides, such as Gly-Pro and Pro-Hyp, enhanced skin properties, reducing UVB-induced aging [42].

The results of the present study show that FBBP isolated from industrial waste bones of silver carp could stimulate the metabolism and cell proliferation of fibroblasts and keratinocytes. In addition, FBBP preparation demonstrated cytoprotective activity in an experimental model of UVB-irradiated skin cells, which suggested usefulness in topical formulations intended for skin protection against photoaging. Further, we investigated the effect of optimal concentrations of FBBP (60 and 120 µg/mL) on processes triggered by UVB irradiation, such as intracellular ROS production, level of inflammation and melanogenesis in several in vitro experimental models mimicking those taking place in skin.

### 2.6. Effect of FBBP on Intracellular ROS Production in Skin Cells

**Figure 5:**
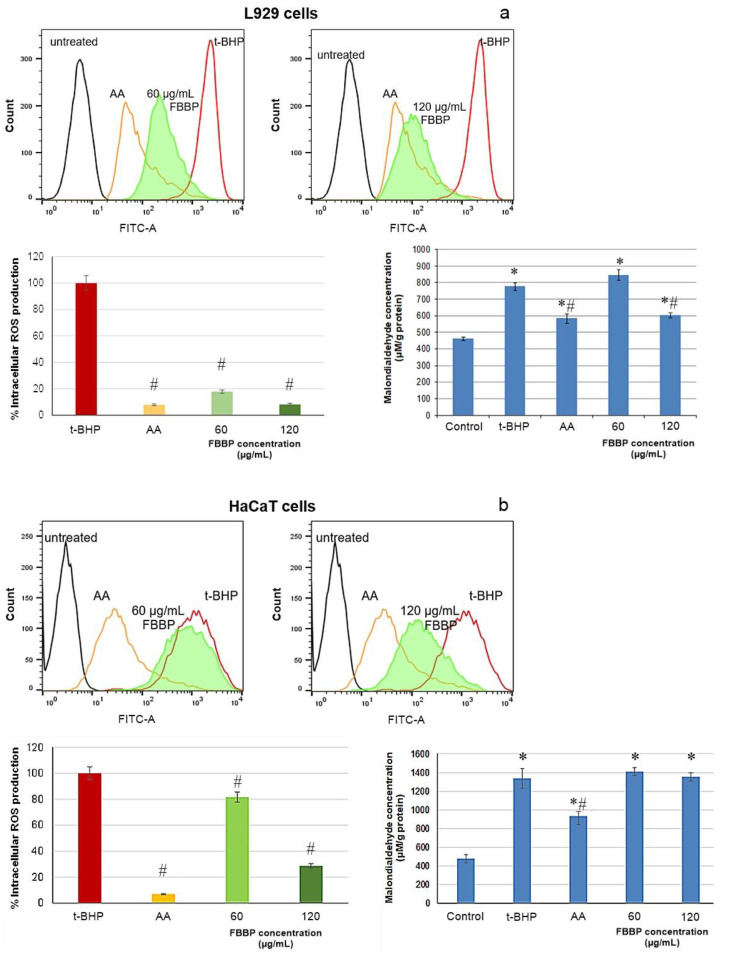
Level of intracellular ROS and MDA production in tert-butyl hydroperoxide (t-BHP) oxidative stress model of L929 (**a**) and HaCaT (**b**) cells treated with different concentrations of FBBP and AA, determined by flow cytometry and TBARS assay, respectively. * *p* < 0.05, compared to untreated control; ^#^ *p* < 0.05, compared to t-BHP-stressed control.

UVB irradiation of skin cells leads to excessive ROS production and, consequently, to damage of biomolecules present in the cell membrane, disturbances in cell metabolism and enzyme secretion [2]. In the present study, FBBP effect on intracellular ROS and malondialdehyde (MDA) production was investigated, for the first time, in both experimental models of oxidative stressed fibroblasts and keratinocytes, in regard of their role as protective agents against oxidative damage and lipid peroxidation. Flow cytometry histograms and the values of intracellular ROS production calculated using Diva software are presented in Figure 5.

The results show that the level of ROS production significantly (*p* < 0.05) decreased down to 8.22% in L929 cells (Figure 5a), while in HaCaT cells, a minimal value of 28.69% was recorded, compared to stressed cells (100%) (Figure 5b). In addition, the ROS production values decreased in a dose-dependent manner in L929 cells and the level recorded at a concentration of 120 µg/mL FBBP (8.22%) was similar to that registered for AA treatment (7.81%), a known antioxidant agent used in cosmetic products. A slower decrease was observed in HaCaT cells, indicating a weaker antioxidant effect of the tested concentrations of FBBP.

The same model was used to evaluate FBBP capacity to prevent lipid peroxidation chain reaction using thiobarbituric acid reactive substances (TBARS) assay. The results show that oxidative stress has induced a significant (*p* < 0.05) increase of intracellular MDA concentration in L929 cells from 461 to 777 µM/g protein, indicating lipid fragments generation (Figure 5a). Pretreatment of L929 cells with 60 µg/mL FBBP could not hinder the process, but, at a concentration of 120 µg/mL FBBP, a significant (*p* < 0.05) decrease of MDA concentration (603 µM/g protein) was registered, close to the value registered in case of AA treatment (584 µM/g protein). In HaCaT cells, high MDA level was observed in FBBP-treated similar to that in oxidative stressed cells, indicating low protection of keratinocytes (Figure 5b).

Exposure to UV light, as an exogenous factor, and the limited endogenous antioxidant system lead to increased intracellular level of ROS and reactive nitrogen species involved in redox signaling and oxidative stress in skin cells [43]. Therefore, the mechanism of photochemical formation of oxidizing agents (hydrogen peroxide-H_2_O_2_, HO radical, superoxide anion, singlet oxygen and peroxynitrite), which react with polyunsaturated fatty acids, consists of a cascade of lipid peroxidation reactions resulting in the formation of lipid hydroperoxides, followed by their fragmentation into reactive aldehydes with impact on altering proteomic and genomic targets and, finally, producing cellular lesions (cell membrane) or cell death by apoptosis and necrosis [44].

Photoprotection and anti-aging cosmeceuticals based on natural antioxidant compounds have recently been developed to prevent skin damage by topical application or oral administration. Several studies demonstrated that fish protein hydrolysates isolated mainly from skin and muscle could represent potential sources of antioxidant bioactive peptides [45], but few analyzed fish bone peptides effect on ROS cellular level in skin cells (fibroblasts and keratinocytes). A recent study reported that fish collagen peptides isolated from redlip croaker scales by neutrase digestion, having a MW of 733–886 Da, allowed the decrease of ROS level in oxidative damaged HepG2 hepatocyte carcinoma cells [46]. The results are similar to those found in our study, indicating the antioxidant activity of fish bone peptides is as a result of their reaction with ROS species based on a hydrogen atom transfer mechanism.

In regard to the fish peptides’ effect on lipid peroxidation, the same study reported their capacity to significantly reduce the MDA level in oxidative stressed HepG2 cells, indicating their protection role by lowering the oxidative stress injury [46]. In vivo studies on mice indicated a slight increase of MDA plasma level within the physiological range of concentrations after oral administration of marine collagen peptides from deep sea fish skin, but this was discussed as beneficial for the stimulation of collagen synthesis, adenosine triphosphate (ATP) storage and sebum production, in view of skin properties improvement [23]. Other studies reported that gelatin peptides obtained from salmon skin enabled significant decrease of plasma and skin level of MDA in UV-irradiated mice, indicating their capacity to inhibit lipid peroxidation and to repair photoaged skin [47]. Oral administration of collagen hydrolysate obtained from carp skin improved collagen, hyaluronic acid and antioxidative activity in UV-photoaged mice, in correlation to their MW decrease [13].

All these data indicate that FBBP isolated in the present study showed the capacity to intervene in redox processes by reduction of the oxidative stress in skin cell cultures, especially in fibroblasts, which took place through significant decrease of the ROS level and, to a lesser extent, of the lipid peroxidation. Thus, FBBP could represent natural antioxidant ingredients of cosmeceutical formulations, ensuring photoprotection in stressed skin cells.

### 2.7. Effect of FBBP on Pro-Inflammatory Cytokines Production in THP-1-Derived Macrophages

**Figure 6:**
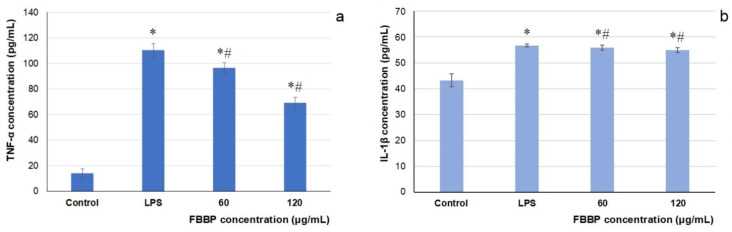
Secretion of TNF-α (**a**) and IL-1β (**b**) pro-inflammatory cytokines in LPS-inflamed THP-1 macrophages pretreated with different concentrations of FBBP, determined by ELISA assay at 24 h of cultivation. * *p* < 0.05, compared to untreated cells (control); ^#^ *p* < 0.05, compared to LPS-inflamed cells.

In the present study, THP-1-derived macrophages inflamed by lipopolysaccharide (LPS) treatment served as in vitro experimental model for the evaluation of the anti-inflammatory activity of FBBP preparation. The level of TNF-α and interleukin 1 beta (IL-1β) pro-inflammatory cytokines secretion in LPS-stimulated macrophages cultivated in the presence of FBBP was determined by enzyme-linked immunosorbent assay (ELISA). The results show that LPS treatment significantly (*p* < 0.05) increased TNF-α and IL-1β production by 770% and 155%, respectively, compared to the untreated control (Figure 6). In response to FBBP treatment, the level of TNF-α pro-inflammatory cytokine significantly (*p* < 0.05) decreased in a dose-dependent manner by 15% and 40%, at increasing concentrations of 60 and 120 µg/mL, respectively (Figure 6a). In turn, IL-1β level was significantly (*p* < 0.05) lowered by FBBP treatment, compared to the inflamed control, but in a range of 18–20% (Figure 6b).

Skin exposure to UV radiation, primarily to UVB wavelengths, could induce erythema and inflammatory response in epidermis and upper dermis, as documented by histological observations [48]. The cascade of inflammatory mediators’ synthesis and ROS formation and Langerhans cells decrease define the mechanisms leading to sunburn, in short exposure, or skin photoaging and carcinogenesis, in chronic exposure, triggering the innate immune response [49]. The experimental model used in the present study was based on LPS-inflamed macrophages, knowing that LPS component of the outer membrane of Gram-negative bacteria could act as an endotoxin, inducing septic shock syndrome and stimulating the synthesis of inflammatory mediators, such as pro-inflammatory cytokines (TNF-α, IL-1β and IL-6), nitric oxide, prostanoids and leukotrienes. TNF-α is the main cytokine involved in systemic inflammation, especially in the acute phase reaction, with an important role in immune cells regulation, while IL-1β is involved in cell proliferation, differentiation and apoptosis. In previous studies, LPS was recognized by macrophage cells as a pathogen-associated molecular pattern and involved in upregulation of cell surface Toll-like receptor 4 (TLR4) after specific binding [50]. The internalization of TLR4 in endosomes led to activation of the immune response via TIR-domain-containing adapter-inducing interferon-β (TRIF)-dependent signaling pathway, controlled by CD14 membrane protein [51]. Previous reports showed that peptides could inhibit TLR4 endocytosis and the triggered cascade signaling pathways via phosphorylation of mitogen-activated protein kinases (MAPK) and nuclear translocation of nuclear factor kB (NF-kB) pathway components in LPS-stimulated RAW264.7 cells [52]. An antioxidant peptide isolated from *O. livida* secretion inhibited in vivo erythema inflammation in UVB-irradiated mice skin, providing photoaging potential [39].

The present study offered new scientific data that demonstrated, for the first time, the anti-inflammatory activity of FBBP in LPS-treated THP-1-derived macrophage cells by significantly attenuation of pro-inflammatory cytokines production, especially of TNF-α level, which could offer protection against inflammatory response in UVB-exposed skin.

### 2.8. Effect of FBBP on Melanogenesis in UVB-Irradiated Mel-Juso Cells

**Figure 7:**
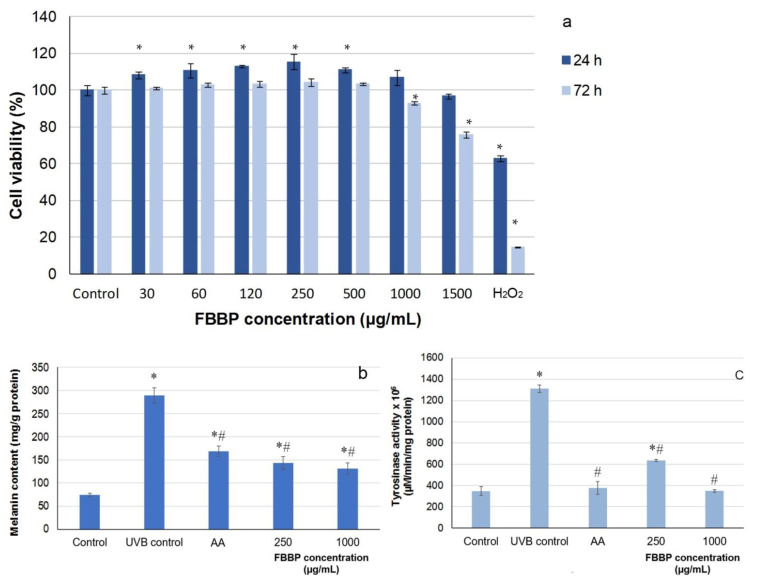
Cell viability of Mel-Juso melanoma cells cultivated in the presence of different concentrations of FBBP, for 24 and 72 h, determined by MTT assay (**a**). The effect of FBBP and AA on melanin content (**b**) and tyrosinase activity (**c**) in UVB-irradiated Mel-Juso melanoma cells. * *p* < 0.05, compared to untreated control; ^#^ *p* < 0.05, compared to UVB control.

In the present study, the cytocompatibility of FBBP in Mel-Juso melanoma cells was first evaluated by MTT assay. The results show that FBBP were non-cytotoxic (cell viability >80%) on the entire tested range of concentrations (30–1500 µg/mL) at 24 h of cultivation (Figure 7a). At 72 h of cultivation, FBBP were cytocompatible in the same range of concentrations, except for the concentration of 1500 µg/mL, which decreased the cell viability to 75%. Two concentrations of FBBP were selected for further testing, 250 µg/mL giving the highest cell viability (118%) and 1000 µg/mL as the highest cytocompatible value.

In the model of UVB-irradiated Mel-Juso cells, the intracellular melanin secretion was stimulated by 3.9-fold after three-day exposure to UVB radiation (Figure 7b). The effect of FBBP pretreatment on the suppression of UVB-induced melanin synthesis was determined by melanin production measurement. Thus, the melanin content significantly (*p* < 0.05) decreased by 51% and ~55% at concentrations of 250 and 1000 µg/mL FBBP, respectively, compared to UV-treated control (Figure 7b). Similarly, AA treatment lowered the melanin secretion by 42%. In addition, FBBP treatment significantly (*p* < 0.05) inhibited tyrosinase activity by 52% at a concentration of 250 µg/mL and 74% at 1000 µg/mL, reaching the normal level of control cells, also observed in AA-treated cells (Figure 7c).

Antioxidants, such as AA, hydroquinone, arbutin or niacinamide, were used as melanogenesis inhibitory agents, but safety was discussed related to the used concentrations [53]. It was previously reported that certain peptides containing Arg in combination with Ala, Val or Leu were strongly bound to tyrosinase and inhibited its key activity in melanin production process [54]. This observation could also explain the downregulation of melanogenesis by peptides from FBBP preparation obtained in the present study by papain digestion. In vivo studies examined oral administration of fish scale collagen peptides to women and reported their ability to decrease the area of UV spots [55]. Melanocytes response to skin inflammation after UVB exposure can occur through hyperpigmentation, especially on the face, creating solar lentigo (sun spots) and causing distress to the affected individuals [56]. Recently, melanogenesis process was showed to be associated with innate immune response level, sharing the same TLR modulation and MAPK signaling pathways discussed in relation to the anti-inflammatory activity [57]. These mechanisms could probably explain the relevant findings in the present study showing the capacity of FBBP to suppress cellular melanin biosynthesis in UVB-exposed cells and their depigmenting effect, similar to that of AA. In this context, FBBP could be further tested as natural depigmentation compounds and potential use in photoprotective formulations.

Due to all these beneficial properties verified within different in vitro experimental models, FBBP preparation might be useful as bioactive ingredient to develop novel cosmeceuticals intended to exert both cosmetic and therapeutic effect on skin cells function during UV exposure.

### 2.9. Separation of FBBP Fractions and Identification of Antioxidant Peptides

**Figure 8:**
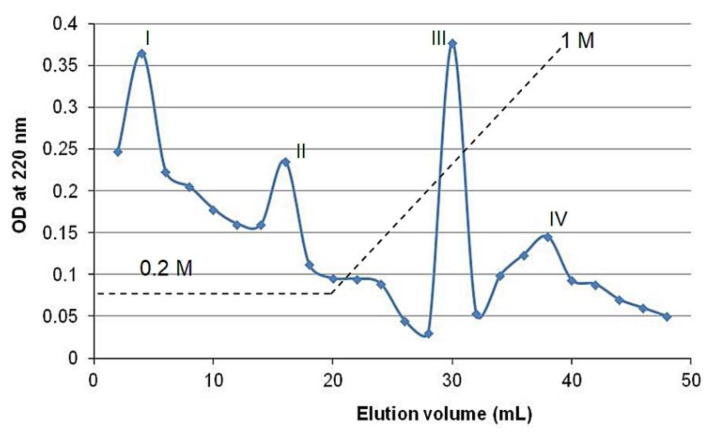
Ion exchange chromatography of FBBP on CM-Sephadex using 0.2–1 M NaCl gradient.

During cation exchange chromatography, FBBP preparation was separated into four peaks (I–IV), two collected at low ionic strength and two during the gradient elution with increasing NaCl concentrations (0.2–1 M) (Figure 8), indicating the presence of peptides with variable pI. Thus, peptides with low pI values carried a net negative charge at pH 7 and eluted first, followed by those with pI close to 7, while peptides with high pI values carried a net positive charge, bound to the resin and eluted at high salt concentration.

In addition, the fractions were analyzed for their capacity to scavenge synthetic free radicals of 2,2′-azino-bis(3-ethylbenzothiazoline-6-sulfonic acid) (ABTS) and hydroxyl (HO) radicals also present in the biological environment. The results showed that peptides from Fractions III and IV had significantly (*p* < 0.05) higher antiradical activities, compared to those of peptides from Fractions I and II, in both experimental models (Table 2). Similar studies analyzed and reported the antioxidant activity of various enzymatic fish bone hydrolysates, excepting silver carp (Table 1).

Fourteen bioactive peptides were identified in FBBP preparation, in a previous study, by matching the peaks of matrix assisted laser desorption ionization–time of flight (MALDI-TOF) mass spectrum, corresponding to 0–3000 mass-to-charge ratios (*m*/*z*), and the amino acid sequences provided by simulated papain cleavage of type I collagen, the most abundant protein in bone [17]. In the present study, we correlated the calculated MW and isoelectric point (pI) values of the collagenic peptides ranging 0–2000 *m*/*z* and presenting the characteristic -Gly-X-Y- repetitive sequence, to the chromatography data, in particular the ion exchange and RP-HPLC results, as well as to their antiradical activity. Five collagenic bioactive peptides consisting of 9–21 amino acid residues were identified and presented the following properties: MW ranging 817.81–1874.01 Da and pI ranging 4.37–9.51. The peptides sequences are given in Table 2, according to their increasing pI and likely elution during ion exchange chromatography. Acidic amino acids (Glu-E, Asp-D) frequently appeared after Gly residue (G) in the hydrophobic sequences of Pro (P), Ala and Val, while basic amino acids (Arg-R and Lys-K) were found at the C-terminus of peptides. This was in accordance with the documented observation that papain cleaves the protein chain at Arg or Lys residues that are preceded by a hydrophobic amino acid from the following Ala (A), Val (V), Leu (L), Ile (I), Phe (F), Trp (W) and Tyr (Y), but does not cleave the peptide bonds formed by Arg or Lys residues followed by Val residues [58]. The peptides corresponding to Fractions III and IV had the sequences GHRGFSGLDGAK (1201.31 Da) and GEPGAAGGRGPPGERGAPGAR (1874.01 Da), respectively, and presented the highest antioxidant activity.

All these analyses have shown for the first time that papain enzymatic hydrolysis of *H. molitrix* bone waste promoted the formation of antioxidant peptides with low MW consisting mainly of hydrophobic, collagen-characteristic and charged (acidic and basic) amino acids.

The antioxidant activity of fish peptides has lately been discussed in relationship to their amino acid composition and the derived hydrophilic/hydrophobic character, which played an important role in their bioactivity [59]. Thus, Gly residue was frequently encountered in collagen molecule, as every third amino acid (33% of its composition), providing great flexibility to peptide skeleton and the ability to quench unpaired radicals or electrons through the hydrogen atom [60]. Moreover, the high content of hydrophobic amino acids, such as Pro, Ala, Leu, Ile and Val, in collagen-derived peptides was reported to enhance the radical scavenging activity [61]. Hydrophobic sequences could also interact with fatty acid chains, increase their lipid solubility and accessibility to hydrophobic sites, thus intervening in the complex process of lipid peroxidation [62]. Other hydrophobic amino acids with aromatic side chain, such as Tyr, Phe and Trp, and aliphatic side chain, such as Met, could also regulate this process due to their metal chelating capacity, but they were poorly found in collagen molecule [63]. In turn, hydrophilic peptides containing acidic and basic amino acids with electrically charged side groups (COO^−^ and NH_3_^+^) had increased solubility and free radicals quenching ability due to excess of electrons. In previous studies, hydrophilic peptides also showed efficiency in Fe^2+^ ions binding and decrease of metal ions pro-oxidative capacity in ROS formation systems [64].

The role of specific amino acids position for increased antioxidant activity of food-derived peptides has recently been reported [65]. Thus, C-terminal Arg residue in peptides from seabass skin hydrolysates was found highly correlated to ABTS free radical scavenging capacity, similar to peptides from the present study, while N-terminal Cys (C) improved HO radical scavenging, compared to similar synthetic tripeptides with sequence permutation. Other studies found that Val or Leu end location, corroborated with conformational characteristics of the peptide molecules containing Pro, His or Tyr, increased the potential to act as antioxidant agents [66].

Besides peptides composition and sequence, their size was reported as an important parameter related to the antioxidant activity [67]. It was showed that low MW bioactive peptides of 2–10 amino acids have exerted better ROS scavenging activities, compared to those of polypeptides from which they derived because the small size favored a more efficient interaction with free radicals [61].

All these data indicate that peptidic fractions isolated from silver carp bones and the identified bioactive peptides with specific composition, sequence and amphiphilic character might act synergically with organic/inorganic UV filters from sunscreens to complement UV absorption with antiradical activity.

## 3. Materials and Methods

### 3.1. Preparation of Bioactive Peptides from Fish Bone Tissue

Silver carp headless frames were supplied on ice by a local fishery (Tulcea, Romania). The preparation of bioactive peptides was performed by papain treatment and centrifugal ultrafiltration. Briefly, the bone tissue (200 g) was minced and homogenized with distilled water, in a weight ratio of 1:1, using a knife homogenizer. Then, bone tissue decalcification was performed in a solution of 1% ethylene glycol-bis(β-aminoethyl ether)-N,N,N’,N’-tetraacetic acid (EGTA) in 0.05 M Tris buffer, pH 7 by stirring at room temperature for 8 h and centrifugation at 10,000× *g* for 30 min. For delipidation, the paste was mixed with 200 mL of 0.05 M Tris buffer and incubated at 55 °C for 4 h and at 90 °C for 15 min. The mixture was kept at 4 °C overnight and then centrifuged at 10,000× *g* for 20 min to remove the upper layer.

Enzymatic hydrolysis was carried out in a water bath by paste incubation in 4% papain solution, pH 5.5, in a weight ratio of 25:1, at 55 °C, for 6 h. At the end of the incubation, the solution was heated at 100 °C for 5 min to inactivate the enzyme. Subsequently, the solution was cooled at room temperature and centrifuged at 10,000× *g* for 30 min. The supernatant representing a solution of FBH was subjected to centrifugal ultrafiltration at 7500× *g*, for 25 min using filter units with cellulose membranes of 3000 Da MW cutoff (Amicon, Merck, Darmstadt, Germany). The isolated permeate representing the solution of FBBP was partly stored at −20 °C until analysis and partly concentrated in a rotary evaporator (Laborota-4000, Heidolph, Schwabach, Germany) and then lyophilized in a Christ freeze-dryer (Germany), yielding a yellowish powder. The protein content was determined by bicinchoninic acid (BCA) assay using a standard curve built with gelatin type A (Sigma) as standard protein (0–2 mg/mL).

### 3.2. Determination of MW Distribution

#### 3.2.1. Size Exclusion Chromatography

Samples of FBH and FBBP were loaded onto a Sephadex G-75 column (1 × 27 cm) and the elution was performed with distilled water, pH 7, at a flow rate of 0.5 mL/min. Fractions of 2 mL were collected and the optical density (OD) was recorded at 220 nm using a V650 UV-VIS spectrophotometer (Jasco, Tokyo, Japan). The high absorbance fractions were pooled, lyophilized and analyzed by ion exchange chromatography. Standards of known MW (bacitracin, insulin, cytochrome c and albumin) were eluted in the same conditions to build a calibration curve.

#### 3.2.2. RP-HPLC Analysis

RP-HPLC analysis of FBBP sample was performed on an Agilent 1200 HPLC system equipped with quaternary pump, thermostated autosampler and diode array detector (Agilent, Santa Clara, CA, USA), using a Europa C18 peptide column (4.6 mm i.d. × 150 mm length), 120 Å (Teknokroma, Barcelona, Spain). A volume of 10 μL sample was injected and the elution was carried out in a mobile phase (A, pure water containing 0.1% trifluoroacetic acid; B, acetonitrile containing 0.08% trifluoroacetic acid) using a linear gradient of 0–60% B at a flow rate of 1 mL/min and temperature of 37 °C for 30 min. The OD was measured at 218 and 280 nm.

### 3.3. Determination of UV Absorption Factor

UV absorption capacity of peptide sample was evaluated by UV spectroscopy, according to “UV shield factor calculation” software (Jasco, Japan) instructions. Lyophilized sample (6 mg) was suspended in 1 mL of distilled water and the UV spectra of serially diluted sample were recorded at a V-650 spectrophotometer (Jasco, Japan). The UV absorption factor was calculated by reporting to a default value of 100 on each UVA, UVB and UVC domain using the dedicated software. A control of commercial SPF 6 lotion was similarly analyzed. The samples were analyzed in triplicate.

### 3.4. In Vitro Cytocompatibility in Normal and Irradiated Skin Cells

#### 3.4.1. In Vitro Cytocompatibility Model in Normal Cells

Mouse fibroblasts from NCTC clone L929 cell line (ECACC) were seeded at a density of 4 × 10^4^ cells/mL in 96-well plates and cultured in Minimum Essential Medium (MEM) supplemented with 10% fetal bovine serum (FBS) and 1% mixture of antibiotics (penicillin-streptomycin-neomycin-PSN), at 37 °C in 5% CO_2_ humid atmosphere, for 24 h. Human keratinocytes from HaCaT cell line (ECACC) were seeded at a density of 5 × 10^4^ cells/mL in 96-well plates and cultured in Roswell Park Memorial Institute (RPMI) 1640 supplemented with 10% FBS and 1% PSN, in standard conditions, for 24 h. For the experiments, the culture medium was replaced with fresh culture medium supplemented with FBS, containing FBBP concentrations ranging 10–1500 µg/mL and cell cultivation continued in standard conditions, for 48 h. Cells cultivated in the culture medium without sample served as negative control, while cells treated with 10 µg/mL H_2_O_2_ served as positive control that induced cell necrosis. Cells treated with 15 µg/mL AA, a known antioxidant used in cosmetics was used as control with stimulative effect on cell proliferation. The experiments were performed in triplicate.

#### 3.4.2. In Vitro Cytoprotective Model in UVB-Irradiated Cells

**Figure 9:**
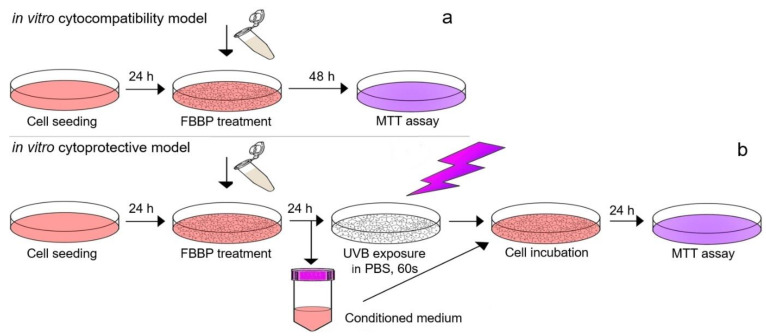
Schematic representation of in vitro experimental models developed for FBBP cytocompatibility testing in normal conditions (**a**) and cytoprotection in UVB-irradiated cells (**b**).

For the irradiation experiment, the same cell cultures (L929 and HaCaT) and treatment protocol were performed as described above but using a density of 1 × 10^5^ cells/mL in 35 mm Petri dishes. After FBBP treatment for 24 h, the cells were irradiated using a VL-6 LM UV lamp with a maximum energy at 312 nm (UVB range) for 60 s (127 mJ/cm^2^). Before UV exposure, the culture medium was harvested, replaced with PBS and irradiation was performed in the laminar hood to preserve cell sterility and to avoid overheating. After irradiation, the harvested medium containing FBBP was added over the cells and cultivation continued for 24 h in standard conditions. The cell viability was evaluated by MTT assay, as described above. The experiments were performed in triplicate. Scheme of in vitro experimental models of FBBP cytocompatibility in normal conditions and cytoprotection in UVB-irradiated cells is presented in Figure 9.

#### 3.4.3. Cell Viability Assay

MTT assay was used for FBBP cytocompatibility testing according to the international standard ISO 10993-5/2009 based on the reaction of yellow MTT with mitochondrial dehydrogenases from the metabolically active cells through dihydronicotinamide adenine dincleotide (NADH) reduction and formation of purple insoluble formazan crystals, as previously described [68]. At the end of cells incubation, the culture medium was replaced with 100 μL MTT solution in MEM (0.25 mg/mL) and the plate was incubated in 5% CO_2_ atmosphere, at 37 °C, for 3 h. Then, the MTT solution was replaced with isopropyl alcohol to solubilize the formazan crystals by gentle shaking, for 15 min. The OD was measured in each well at a wavelength of 570 nm using a Spectrostar nano microplate reader (BMG Labtech, Germany). The values were directly proportional to the number of viable cells.

#### 3.4.4. Cell Morphology Observations by Light Microscopy

Cell morphology of FBBP-treated cultures with and without UVB irradiation was observed in a parallel experiment, in the same conditions as described above. At the end of the incubation, the cells were examined and phase contrast micrographs were acquired at an AxioStar Plus microscope equipped with a digital camera (Carl Zeiss, Jena, Germany).

### 3.5. Determination of Antioxidant Activity in Skin Cell Cultures

#### 3.5.1. Determination of Intracellular ROS Production

The effect of FBBP on intracellular ROS production has been evaluated in an experimental model of oxidative stress induced in skin cells culture, as previously described [69]. L929 cells were seeded at a density of 5 × 10^4^ cells/mL and HaCaT cells at a density of 6 × 10^4^ cells/mL in 12-well culture plates and cultivated in MEM, in standard conditions, for 24 h. Adhered cells were incubated with fresh medium containing 60 and 120 µg/mL FBBP, respectively, at 37 °C in humidified atmosphere with 5% CO_2_ for 24 h, and, then, cells were treated with 50 µM t-BHP, for 30 min.

The cell permeant fluorogenic dye 2′,7′-dichlorofluorescin diacetate (DCFH-DA) was used to measure ROS production at cellular level. Upon reaction with free radicals, formation of DCF fluorescent product was monitored. The cells treated as above were incubated with 10 µM DCFH-DA for 30 min and analyzed using BD LSR II flow cytometer (Becton Dickinson, Franklin Lakes, NJ, USA). Acquired histograms were processed to calculate ROS production (%) in correlation to the fluorescence intensity using FlowJo and Diva software. Cells incubated in normal medium and cells pre-treated with 12 µM AA were processed in similar conditions and served as controls.

#### 3.5.2. Determination of Lipid Peroxidation by TBARS Assay

Using the same model of oxidative stressed skin cells treated with FBBP as described above, the level of lipid peroxidation was analyzed by determination of MDA production using TBARS spectrophotometric assay, as previously described [70]. Thus, at the end of the incubation with oxidizing agent, the cells were lysed and the supernatant was mixed with thiobarbituric acid (TBA) reagent, in acid medium, at 95 °C, for 1 h. After centrifugation at 3000× *g*, for 10 min, the OD of the mixtures containing the MDA-TBA adduct was recorded at 532 nm using a V-650 UV-VIS spectrophotometer (Jasco). The lysate was also analyzed for protein content by the BCA method. The results were expressed in µM MDA/g protein, considering ɛ = 1.55 × 10^6^ M^−1^ cm^−1^ for the MDA-TBA complex.

### 3.6. Determination of In Vitro Anti-Inflammatory Activity

The anti-inflammatory activity was evaluated in an experimental model of macrophages derived from human THP-1 leukemic monocyte cells (ATCC), as previously described [71]. Briefly, cells were seeded at a cell density of 1 × 10^6^ cells/mL in 24-well plates in RPMI 1640 medium with high glucose content (4.5 g/L), supplemented with 10% FBS and 1% mixture of antibiotics and incubated in humid atmosphere with 5% CO_2_ at 37 °C for 18 h. After cells differentiation in macrophages by treatment with 100 ng/mL 12-myristate 13-acetate (PMA) for 72 h, the culture medium was replaced with fresh medium containing 60 and 120 µg/mL FBBP, respectively, and incubation continued for 1 h. Then, cells were incubated with 10 ng/mL LPS to induce inflammation and incubation continued for 24 h. The culture media were harvested and centrifuged at 400× *g*, for 10 min and TNF-α and IL-1β proinflammatory cytokines production was analyzed using ELISA sandwich kits, according to the manufacturer’s protocol (Invitrogen). Cell viability was evaluated by MTT assay. The results were expressed as pg/mL after normalization to viable cells. The experiments were performed in triplicate.

### 3.7. Analysis of Melanogenesis

#### 3.7.1. Cytocompatibility Assay

The stabilized Mel-JuSo human melanoma cell line was cultured in RPMI culture medium supplemented with 10% FBS, 1% non-essential amino acids, 2 mM glutamine, 1 mM sodium pyruvate and 1% PSN mixture of antibiotics. For experiments, cells were seeded at a density of 4 × 10^4^ cells/mL in 96-well plates and allowed to adhere overnight. Then, the medium was replaced with fresh culture medium containing FBBP concentrations in the range of 30–1500 μg/mL and incubation continued in standard conditions for 24 and 72 h. Cell viability was evaluated by MTT assay, as described above. Untreated cells cultivated in standard conditions served as negative control and cells cultivated with 15 µg/mL H_2_O_2_ served as positive control.

#### 3.7.2. Determination of Melanin Production

Subconfluent cells were trypsinized, seeded at a density of 4 × 10^4^ cells/mL in 35 mm Petri dishes and incubated in a humid atmosphere with 5% CO_2_ for 7 h. Then, 250 and 1000 µg/mL FBBP were added in culture medium, respectively, and incubation continued overnight. Then, cells were irradiated using a VL-6 LM UV lamp at 312 nm, vertically, for 10 s (21 mJ/cm^2^) in three consecutive days. During irradiation, the conditioned medium was replaced with PBS. The intracellular melanin content was measured in cell lysate obtained by treatment with 0.1% Triton X-100 in Tris-HCl buffer pH 7.5 and centrifugation at 3000× *g* and 4 °C for 10 min, as previously described [72]. The supernatant was harvested for protein content analysis by the BCA assay. The precipitate was dissolved in 300 μL 1 N NaOH containing 10% dimethyl sulfoxide by incubation at 80 °C, for 1 h. The OD was measured at 405 nm using a Spectrostar nano microplate reader (BMG Labtech, Ortenberg, Germany) and the values were normalized to the protein concentration. The results were expressed in mg melanin/g protein.

#### 3.7.3. Determination of Tyrosinase Activity

Using the same model of UVB-irradiated melanocytes treated with FBBP as described above, the cellular tyrosinase activity was evaluated by measuring the 3,4-dihydroxy-l-phenylalanine (L-DOPA) oxidation rate [73]. At the end of the incubation period, cells were lysed and the supernatant was mixed with 2 mg/mL L-DOPA solution and incubated at 37 °C. The OD was registered at 475 nm at every 2 min, for 30 min using a Spectrostar nano microplate reader (BMG Labtech, Germany). Lysis buffer was used as control. The results were calculated as µM/min and were normalized to the protein concentration determined by the BCA assay.

### 3.8. Separation of FBBP Fractions and Antioxidative Peptides Identification

#### 3.8.1. Ion Exchange Chromatography

FBBP sample was loaded onto a CM-Sephadex C-50 column (1 × 7 cm), equilibrated with 0.2 M NaCl solution. The elution was performed using a volume of 0.2 M NaCl and then, a linear gradient of NaCl in the range of concentrations 0.2–1 M, at a flow rate of 1 mL/min. Fractions of 3 mL were collected and the OD was read at 220 nm using a V650 UV-VIS spectrophotometer (Jasco, Japan). The high absorbance fractions were pooled, lyophilized and analyzed for antioxidant activity.

#### 3.8.2. ABTS Free Radicals Scavenging Analysis

The capacity to scavenge ABTS free radicals was evaluated by Trolox equivalent antioxidant capacity (TEAC) assay [74]. Briefly, the stock solution of 7 mM ABTS containing 2.45 mM potassium persulfate was diluted to reach an OD value of 0.7 ± 0.02 at 734 nm (control). Diluted samples (100 µL) were incubated with ABTS reagent (1 mL) at room temperature in the dark for 10 min. Then, OD of the reaction mixtures was recorded at V-650 UV-VIS spectrophotometer (Jasco, Japan). The percentage of ABTS radical inhibition was calculated using the following formula:

ABTS radicals inhibition (%) = (OD_control_ − OD_sample_)/OD_control_ × 100 (1)

A calibration curve was built using Trolox, an analog of vitamin E with known antioxidant activity, in the range of concentrations of 0–150 µM. The antioxidant activity was expressed as mM Trolox equiv/g protein. All samples were analyzed in triplicate.

#### 3.8.3. HO Radicals Scavenging Analysis

The capacity to scavenge HO radicals generated by Fenton reaction was determined in a microplate assay, as previously described [75]. Diluted sample (80 µL) was mixed with 2 mM 1,10 phenanthroline (40 µL) in the wells of a 96-well microplate. Then, 40 µL of each 2 mM FeSO_4_ solution and 0.03% (*v*/*v*) H_2_O_2_ were added into the mixture. The microplate was incubated at 37 °C in the dark for 1 h and the OD of the resulting solution was measured at 536 nm using Spectrostar nano microplate reader (BMG Labtech, Germany). The percentage of HO radical inhibition was calculated using the following formula:

HO radicals inhibition (%) = (OD_sample_ − OD_negative control_)/(OD_control_ − OD_negative control)_ × 100 (2)

where negative control replaced the sample with distilled water and the control was replaced H_2_O_2_ with distilled water.

The calibration curve was built using Trolox in the range of concentrations of 0–500 µM. The antioxidant activity was expressed as mM Trolox equiv./g protein. All samples were analyzed in triplicate.

#### 3.8.4. Peptides Identification

For the identification of collagenic peptides with high antioxidant activity in FBBP preparation, an analysis of correlation between ion exchange chromatography and free radicals scavenging data obtained as described above was performed; the peptides were isolated by MALDI-ToF mass spectrometry analysis in the range of 0–2000 *m*/*z*; and their amino acid sequences, pI and MW were predicted using Uniprot database information, as previously reported [17].

### 3.9. Statistical Analysis

All experiments were carried out in triplicate. The results were expressed as mean value ± SD for three experiments. Statistical analysis of the data was performed on each pair of interest using two-tailed, paired Student’s *t*-test (Office Excel 2010 software). Differences were considered statistically significant at *p* < 0.05.

## 4. Conclusions

This study offered an efficient processing approach, easy to scale-up for FBBP isolation from discarded freshwater fish bones and a correlation study of their physico-chemically characterization to photoprotective properties, namely UV absorption factor, anti-inflammatory activity and inhibition of intracellular ROS, MDA, melanin production and tyrosinase activity. Evaluation of FBBP in skin cell cultures showed cytocompatibility and cytoprotective properties against UVB-irradiation, good capacity to reduce ROS level and, to a lesser extent, the lipid peroxidation in both L929 fibroblasts and HaCaT keratinocytes. In vitro experiments showed, for the first time, the anti-inflammatory activity of FBBP in LPS-stimulated THP-1-derived macrophages by significantly lowering the TNF-α and IL-1β pro-inflammatory cytokines production. Moreover, FBBP suppressed melanin production in UVB-irradiated Mel-Juso cells through inhibition of tyrosinase activity, a key enzyme involved in melanogenesis. Finally, FBBP fractions with high antiradical activity were separated and identification of collagenic peptides showed sequences of acidic amino acids alternating with hydrophobic ones and ending with basic amino acids, having a MW of 1201.31 and 1874.01 Da. All this new scientific evidence strongly suggests that FBBP isolated from discarded *H. molitrix* bones might have an important impact on skin cells metabolism due to their antioxidant, anti-inflammatory and melanin suppression activity. Future work will envisage the importance of fish peptides beneficial properties in relation to in vivo skin photoaging process.

## Figures and Tables

**Table 1:** Antioxidant activity of fish bone peptides.

Source	Enzyme	Sequence (MW)	Antioxidant Activity
Hoki (*Johnius belengerii*) frame protein	Pepsin, trypsin, papain, a-chymotrypsin, alcalase, neutrase	-ESTVPERTHPACPDF- (1801 Da)	2,2-diphenyl-1-picrylhydrazyl (DPPH), HO, superoxide, peroxyl radical scavenging activity Lipid peroxidation inhibition [30]
Tuna (*Thunnus orientalis*) backbone	Alcalase, α-chymotrypsin, neutrase, papain, pepsin, trypsin	-VKAGFAWTANQQLS- (1519 Da)	DPPH, HO, superoxide radical scavenging activity, Lipid peroxidation inhibition [31]
Atlantic cod (*Gadus morhua*) backbone	Bacterial protease (Protamex)	-WMDF-	DPPH radical scavenging activity [32]
Salmon (*Salmo salar*) backbone	Bacterial endopeptidase (Corolase^®®^ 7089), bacterial protease (Protamex^®®^), papain, bromelain, trypsin	-	DPPH radical scavenging activity [33]
Skipjack tuna (*Katsuwonus pelamis*) bone	Trypsin, chymotrypsin	-SSGPPVPGPMGPMGPR- (1520 Da)	DPPH, ABTS, superoxide radical scavenging activity [34]
Snapper fish (*Lutjanus campechanus*) scales	Alcalase	<5000 Da	ABTS, HO radical scavenging activity [35]

**Table 2:** The antiradical activity of ion exchange chromatography fractions of bioactive peptides from *H. molitrix* bones determined against ABTS and HO radicals. The sequence, molecular weight (MW) and pI of peptides identified in the UniProt database matching the mass spectrum peaks. Reprinted from ref. [17].

Sample	ABTS Scavenging Activity (mM Trolox Equiv./g Protein)	HO Scavenging Activity (mM Trolox Equiv./g Protein)	Peptide Sequence	MW (Da)	pI
Fraction I	2.85 ± 0.78	18.68 ± 1.06	812-GEAGDNGAK-820	817.81	4.37
Fraction II	2.73 ± 0.14	15.38 ± 1.84	337-GEVGPQGAR-345	869.93	6.00
			423-GPPGDAGRAGEPGLVGAR-440	1633.78	6.07
Fraction III	57.15 ± 3.53 *^,#^	294.54 ± 10.48 *^,#^	251-GHRGFSGLDGAK-262	1201.31	8.75
Fraction IV	16.55 ± 1.95 *^,#^	80.37 ± 6.34 *^,#^	458-GEPGAAGGRGPPGERGAPGAR-478	1874.01	9.51

The values are expressed as mean ± standard deviation (SD) (*n* = 3). * *p* < 0.05, compared to fraction I; ^#^ *p* < 0.05, compared to fraction II.

## Data Availability

The data presented in this study are available in this article.

## Supplementary Materials

The following is available online. Figure S1: Spectra of fish bone bioactive peptides (FBBP) preparation isolated from *H. molitrix*, at different concentrations (**a**) and commercial SPF6 lotion (control) (**b**) in UVA, UVB and UVC domains.
