# Supplementary figures and images for: Effect of Fish Bone Bioactive Peptides on Oxidative, Inflammatory and Pigmentation Processes Triggered by UVB Irradiation in Skin Cells
Source: Molecules. 2021 May 4;26(9):2691. doi: 10.3390/molecules26092691 (PMC8124703; doi:10.3390/molecules26092691)

Figure S1

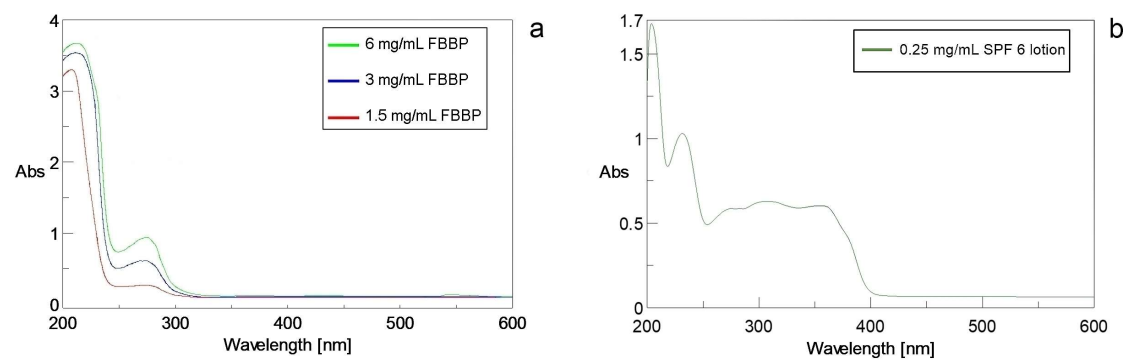

Supplement: Supplementary file 1 [file molecules-26-02691-s001.zip › molecules-1204432-supplementary.pdf]
